# MUSEUMS AS A NETWORK REPOSITORY OF LATE-LIFE CREATIVITY

**DOI:** 10.1093/geroni/igz038.1320

**Published:** 2019-11-08

**Authors:** Manfred Gogol

**Affiliations:** Heidelberg University, Heidelberg, Germany

## Abstract

Through collaboration and exchange for exhibitions, art museums represent an under-explored network for displaying late-life creativity and the longevity dividend. Drawing on the collection from many galleries brought together for the Letzte Bilder (Last Pictures) exhibition by the Schirm Gallery Frankfurt, this talk will illustrate the possibility of creating educational programs, Late Life Creativity trails both virtual through podcasts and structural, as well as specific exhibitions of late-life creativity as a catalyst for reimagining and reframing ageing. Further approaches are the focus on the life course of works of artist like Cy Twombly at the Museum Brandhorst Munich or Pablo Picasso at the Museum Barberini Potsdam, as well as studying aging in artists self-portraits. The interaction of creativity with age, diseases, and functional decline in the museum context can promote the changing of role models and our understanding of productivity and creativity by ourselves as well as in the society.

